# RECIP 1.0 more predictive of overall survival than PSMA PET progression criteria in biochemically recurrent prostate cancer

**DOI:** 10.1007/s00259-025-07592-6

**Published:** 2025-10-28

**Authors:** Kaylee Molin, Jeremy S. L. Ong, Steven Van Der Werf, Roslyn J. Francis, Ghulam Mubashar Hassan, Martin A. Ebert, Jake Kendrick

**Affiliations:** 1 School of Physics, Mathematics and Computing, University of Western Australia, Crawley, WA Australia; 2 Centre for Advanced Technologies in Cancer Research (CATCR), Perth, WA Australia; 3 Department of Nuclear Medicine, Fiona Stanley Hospital, Murdoch, WA Australia; 4 Department of Radiation Oncology, Sir Charles Gairdner Hospital, Nedlands, WA Australia; 5 Department of Nuclear Medicine, Sir Charles Gairdner Hospital, Nedlands, WA Australia; 6 Medical School, University of Western Australia, Crawley, WA Australia; 7 Department of Nuclear Medicine, Royal Brisbane and Women’s Hospital, Brisbane, QLD Australia; 8 Australian Institute for Bioengineering and Nanotechnology, University of Queensland, Brisbane, QLD Australia

**Keywords:** PSMA PET, Biochemically recurrent prostate cancer, Response assessment frameworks, RECIP 1.0, PPP

## Abstract

**Purpose:**

Up to 40% of prostate cancer (PCa) patients experience biochemical recurrence (BCR) after primary treatment, but reliable tools for risk stratification in this setting are limited. This study compared Response Evaluation Criteria in PSMA PET/CT (RECIP 1.0) and PSMA PET Progression (PPP) criteria in predicting overall survival (OS) and prostate-specific antigen progression-free survival (PSA-PFS). As a secondary aim, PROMISE-based nomograms were assessed as tools for OS prediction.

**Methods:**

A cohort of 154 BCR PCa patients with baseline and follow-up [^68^Ga]Ga-PSMA-11 PET/CT was analysed. Disease progression was defined by RECIP 1.0 and three PPP variants (PPP-Volume, PPP-Mean SUV, PPP-Max SUV). Associations with OS and PSA-PFS were tested using Kaplan–Meier and Cox regression models. PROMISE-based visual and quantitative nomograms were applied to follow-up scans to predict OS. Subgroup analyses by treatment type were performed for RECIP and PPP criteria.

**Results:**

RECIP-defined progressive disease (RECIP-PD) was most strongly associated with OS, identifying patients at highest risk of death (median OS 53.2 months; HR 4.62; 95% CI: 2.41–8.87; *p* < 0.001). PPP-based criteria were also prognostic for OS, and were the only framework associated with PSA-PFS. PROMISE-based nomograms were predictive of OS, with performance comparable to PPP criteria. By treatment type, RECIP-PD performed best in androgen deprivation therapy, PPP-Max SUV in radiotherapy, and PPP-Volume in combined ADT + radiotherapy. SUV-based PPP criteria were most predictive of PSA-PFS in combined ADT + radiotherapy.

**Conclusion:**

In BCR PCa, RECIP 1.0 best predicts OS, PPP criteria better predict PSA progression, and PROMISE nomograms provide a single-timepoint approach to OS risk stratification.

**Trial registration number:**

ACTRN ACTRN12615000608561. Registered 11 June 2015. Retrospectively registered.

**Supplementary Information:**

The online version contains supplementary material available at 10.1007/s00259-025-07592-6.

## Introduction

Prostate cancer (PCa) is among the most commonly diagnosed malignancies in men and a leading cause of cancer-related death [1]. Following primary treatment, up to 40% of patients experience biochemical recurrence (BCR), defined by rising prostate-specific antigen (PSA) levels [2, 3]. BCR is clinically important, as it often precedes metastatic progression and is associated with reduced overall survival (OS) [3]. Early identification of patients at highest risk of progression is important for optimising management strategies and improving outcomes.

Current response monitoring in recurrent PCa primarily relies on serum PSA levels and conventional imaging modalities such as CT and bone scintigraphy, as recommended by the Prostate Cancer Working Group 3 (PCWG3) criteria [4]. However, these tools have limited sensitivity and specificity, especially in early-stage disease or in patients with non-PSA-secreting tumours. PSA itself may be an unreliable surrogate, as levels can be affected by treatment effects, tumour heterogeneity, and biological variability [5].

Prostate-specific membrane antigen (PSMA) PET/CT has emerged as a superior imaging modality for detecting PCa recurrence, offering improved sensitivity over conventional techniques [6, 7]. As its clinical use expands, the need for reproducible and standardised PSMA PET–based response criteria has become increasingly apparent. Two response assessment frameworks have been proposed to address this: the PSMA PET Progression (PPP) criteria, which define progression based on new lesions or a ≥ 30% increase in lesion size or tracer uptake [8]; and the Response Evaluation Criteria in PSMA PET/CT (RECIP 1.0), which defines progressive disease (RECIP-PD) as both a ≥ 20% increase in whole-body PSMA tumour volume and the appearance of new lesions [9]. RECIP 1.0 also includes criteria for complete response (RECIP-CR), partial response (RECIP-PR), and stable disease (RECIP-SD), based on changes in PSMA tumour volume and the absence of new lesions. While both frameworks have demonstrated prognostic value in advanced PCa [10–17], their performance in earlier-stage cohorts, such as BCR, remains largely untested.

In a cohort of over 2,000 PCa patients, including those with BCR, Karpinski et al. developed visual and quantitative nomograms for OS-based risk stratification [18]. The visual nomogram is based on tumour count and the presence of metastases in extrapelvic lymph nodes, bones, or organs, while the quantitative model additionally includes total tumour volume, locoregional lymph node involvement, and mean standardised uptake value (SUV). Both models are applied to a single scan and were built using the Prostate Cancer Molecular Imaging Standardized Evaluation (PROMISE) framework, which aims to standardise PSMA PET/CT reporting through a TNM-based system [19]. In the BCR subgroup, the visual and quantitative models showed moderate prognostic performance, with areas under the receiver operating characteristic curve of 0.64 and 0.69, respectively.

The current study aimed to evaluate and compare the prognostic performance of PPP and RECIP 1.0 in patients with BCR PCa. Disease progression was classified using baseline and follow-up [^68^Ga]Ga-PSMA-11 PET/CT scans, and outcomes were assessed for OS and PSA progression-free survival (PSA-PFS). Subgroup analyses were also conducted according to treatment type, including androgen deprivation therapy (ADT) and radiotherapy. As a secondary objective, the visual and quantitative PROMISE-based nomograms were evaluated for their ability to predict OS. By validating these tools in a BCR cohort, this study supports the role of PSMA PET–based classifications in early risk stratification and personalised treatment decisions, including timely intervention for patients at higher risk.

## Methods

### Patient cohort

This study performed a retrospective analysis of 238 patients with BCR PCa who were enrolled in a prospective clinical trial registered with the Australian and New Zealand Clinical Trials Registry (ACTRN12615000608561) [20]. Imaging was performed at either Sir Charles Gairdner Hospital (*n* = 204) or Fiona Stanley Hospital (*n* = 34), in Perth, Western Australia.

Eligibility criteria included BCR following primary treatment of PCa, which was either radical prostatectomy or external beam radiotherapy to the prostate. BCR was indicated by serum PSA levels ≥ 0.2 ng/mL at more than six weeks post-radical prostatectomy or a PSA level 2 ng/mL higher than the previous nadir measurement at three months post-external beam radiotherapy. Patients had to show either negative disease on abdominopelvic contrast CT and bone scintigraphy, or oligometastatic disease with three or fewer lesions.

Following biochemical relapse, patients were treated as per standard clinical care. Treatment approaches included radiotherapy (targeting the prostatic bed, regional lymph nodes, or bone metastases), chemotherapy, ADT, surgery, and/or active surveillance.

The study received ethical approval from the Sir Charles Gairdner Hospital Human Research Ethics Committee (RGS1736) and was conducted in accordance with the principles outlined in the Declaration of Helsinki.

### Scan acquisition

Each patient underwent a baseline [^68^Ga]Ga-PSMA-11 PET/CT scan between June 2015 and July 2016. A dose of 2 MBq/kg of [^68^Ga]Ga-PSMA-11 was administered intravenously, with imaging 60 min post-injection. Imaging was performed using a Siemens Biograph 64 PET/CT scanner at Sir Charles Gairdner Hospital and a Biograph 128 PET/CT scanner at Fiona Stanley Hospital. Prior to imaging, patients were instructed to empty their bladder.

Imaging began with a low-dose CT scan (50 mAs, 120 kVp) for attenuation correction, followed by PET acquisition. PET images were reconstructed with an axial pixel size of 4.07 × 4.07 mm^2^; CT pixel sizes were 0.98 × 0.98 mm^2^ or 1.52 × 1.52 mm^2^, with 2 mm slice thickness for both modalities. The PET scanners were assessed for quantitative accuracy and accredited by the Australasian Radiopharmaceutical Trials Network (ARTnet) [21].

Follow-up scans were performed approximately six months after the baseline scan in cases where the treating physician needed to evaluate disease sites post-treatment. Of the initial 238 patients, 200 underwent follow-up imaging.

### Lesion delineation

Baseline and follow-up scans were segmented semi-automatically. A global SUV threshold of 3 normalised to body weight was applied to the PET images for initial segmentation. These were then reviewed and refined by an experienced nuclear medicine physician (J.O.) using the E-PSMA 5-point system [22]. Only lesions classified as ‘definitely’ or ‘probably’ positive were included. Baseline lesions were carefully matched to corresponding follow-up lesions. Segmentation was performed using MIM Encore software (MIM Software Inc., Cleveland, OH, USA).

### PSMA PET/CT response classification

PPP defines progression as the appearance of one or more new lesions between baseline and follow-up scans or an increase of 30% or more in lesion size or tracer uptake [8]. As the original definition defines progression based on either increases in volume or tracer uptake, we specified three PPP variations to capture both aspects clearly. The first is based on a ≥ 30% increase in lesion volume on the follow-up scan, referred to as ‘PPP-Volume’. To address the part of the definition referring to ≥ 30% increases in tracer uptake, we included ‘PPP-Mean SUV’ and ‘PPP-Max SUV’. Variations in patient size were accounted for by re-normalising SUV values post-segmentation to the mean SUV of a reference volume in the right liver lobe (mean volume: 4.2 cm^3^; range: 3.6–4.8 cm^3^), a method shown to reduce overestimation of SUV in larger patients compared to body weight normalisation [23–25].

**Fig. 1:**
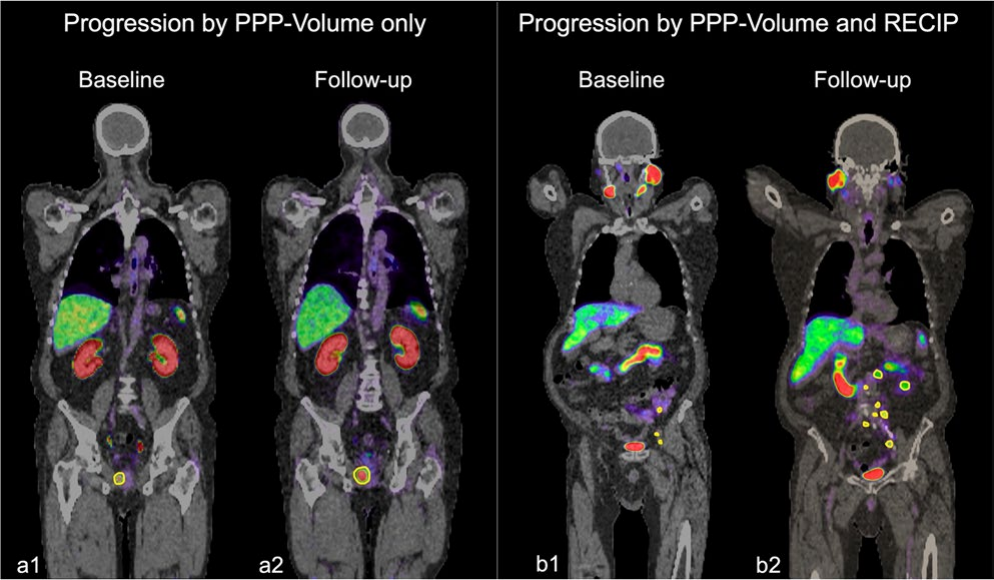
Baseline (a1, b1) and follow-up scans (a2, b2) for Patients A and B, with lesions outlined in yellow. Patient A shows an increase in lesion volume but no new lesions, resulting in progressive disease classification under PPP-Volume only. Patient B demonstrates both new lesions and increased volume, leading to progressive disease classification under both PPP-Volume and RECIP 1.0 criteria

These PPP-based progression criteria were compared to RECIP 1.0, which defines RECIP-PD as a ≥ 20% increase in the total PSMA-positive tumour volume across the whole body, combined with the appearance of one or more new lesions [9]. A key difference between these classifications is that PPP assesses progression on a per-lesion basis—any individual lesion exhibiting a ≥ 30% increase in volume or uptake is sufficient to indicate progression. In contrast, RECIP operates at the patient level and considers only the aggregate tumour volume. Figure 1 illustrates these conceptual differences with example cases classified by PPP-Volume and RECIP-PD.

In addition to RECIP-PD, the RECIP 1.0 framework includes other response categories: partial response (RECIP-PR), complete response (RECIP-CR), and stable disease (RECIP-SD). Definitions of all progression criteria used in the analysis are provided in Table 1.

**Table 1:** Definitions of response frameworks

Framework	Definition
PPP	
PPP-Volume	≥ 30% increase in tumour volume of an existing lesion or the appearance of ≥ 1 new lesion
PPP-Mean SUV	≥ 30% increase in mean tracer uptake in an existing lesion or the appearance of ≥ 1 new lesion
PPP-Max SUV	≥ 30% increase in maximum tracer uptake in an existing lesion or the appearance of ≥ 1 new lesion
RECIP	
RECIP-CR	Complete resolution of tumour uptake on PSMA PET
RECIP-PR	No new lesions and a > 30% reduction in PSMA-positive tumour volume
RECIP-SD	−30% to + 20% change in tumour volume, or ≥ 1 new lesion with a concurrent ≥ 30% reduction, or no new lesions with a ≥ 20% increase
RECIP-PD	Appearance of ≥ 1 new lesion on PET/CT and a ≥ 20% increase in PSMA-positive tumour volume

Finally, the visual and quantitative PROMISE-based nomograms were applied to the follow-up scans to enable comparison with RECIP and PPP, and to assess the potential of single-timepoint imaging for risk stratification. Patients were stratified into high- or low-risk groups using the predefined cut-off values, as outlined in the original publication and its appendices [18].

### Overall survival and PSA progression-free survival

OS was defined as the time from the follow-up scan to death from any cause. Patients who were alive or lost to follow-up were censored at their last recorded clinical encounter, with follow-up ending in March 2023.

PSA levels were collected for each patient from the date of their follow-up scan to their most recent blood test as of July 2024. PSA progression was defined according to the PCWG3 criteria [4] and included death from any cause. For each patient, progression status and the number of days to progression or censoring were recorded.

### Statistical analysis

All analyses were conducted using Python (v3.9.1) and the lifelines package (v0.28.0) [26]. The predictive value of PPP and RECIP 1.0 for OS and PSA-PFS was assessed using Kaplan–Meier curves and Cox proportional hazards models, while PROMISE nomograms were evaluated for OS only. Patients classified as progressive vs. non-progressive under each framework were compared using log-rank tests. Hazard ratios (HRs), concordance indices, and p-values from Cox models quantified associations. A p-value < 0.05 was considered significant.

To assess performance by treatment context, the analyses were repeated in three subgroups: ADT only, radiotherapy only, and both ADT and radiotherapy.

**Fig. 2:**
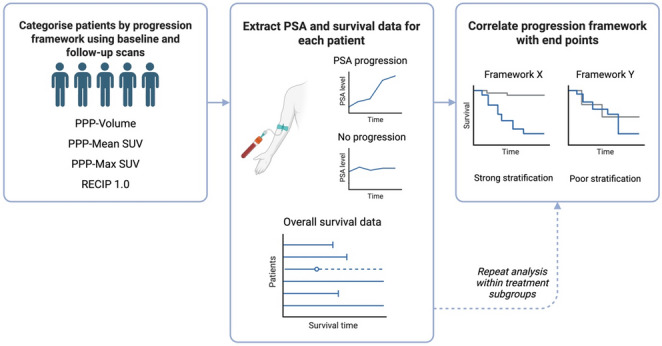
Overview of the methodology. Each patient was classified as having progressive disease or not using each progression framework. The association of each framework with overall survival and PSA progression-free survival was assessed using Kaplan-Meier curves and Cox proportional hazards models

An overview of the analysis pipeline is shown in Fig. 2.

## Results

### Patient characteristics

Of the 238 patients with baseline scans, 200 (84%) underwent follow-up imaging. Among these, 154 (65% of original cohort) patients received interventional therapy and were included in the OS analysis. The median interval between baseline and follow-up scans was 6.2 months (range: 3.2–8.8). Detailed patient characteristics are shown in Table 2.

**Table 2:** Patient characteristics. Continuous variables are presented as median and range; categorical variables are presented as number and percentage

Patient Characteristics (*n* = 154)	
Characteristic	Data
Age (y)	70.1 (45.8–90.0)
Weight (kg)	89 (60–128)
PSA level at referral (ng/mL)	3.2 (0.2–79.5)
Number of lesions at baseline	2 (0–34)
Conventional staging	
0	124 (80.5%)
1	30 (19.5%)
Staging based on PSMA PET/CT scan	
0	21 (13.6%)
1	90 (58.4%)
2	43 (27.9%)
Gleason score*	
< 8	80 (51.9%)
≥ 8	70 (45.5%)
Previous definitive treatment	
Prostatectomy	94 (61.0%)
Radiotherapy	60 (39.0%)
Administered treatment	
ADT	113 (73.4%)
Radiotherapy	74 (48.1%)
Chemotherapy	8 (5.2%)
Surgery	13 (8.4%)
OS status	
Deaths	45 (29.2%)
Survivors	109 (70.8%)
PSA-PFS status^†^	
Progression	64 (65.3%)
No progression	34 (34.7%)

*Gleason score missing for 4 patients. †PSA progression-free survival (PSA-PFS) data were available for 98 patients

For PSA-PFS analysis, only patients with at least three PSA measurements collected after the follow-up scan were included, resulting in a final cohort of 98 patients (41% of the original cohort). The median number of PSA measurements per patient was 10 (IQR: 6–17). Of the 98 patients, 64 (65%) experienced PSA progression, including 30 (31%) who had died by the last follow-up.

### Patient grouping by PPP and RECIP 1.0

**Fig. 3:**
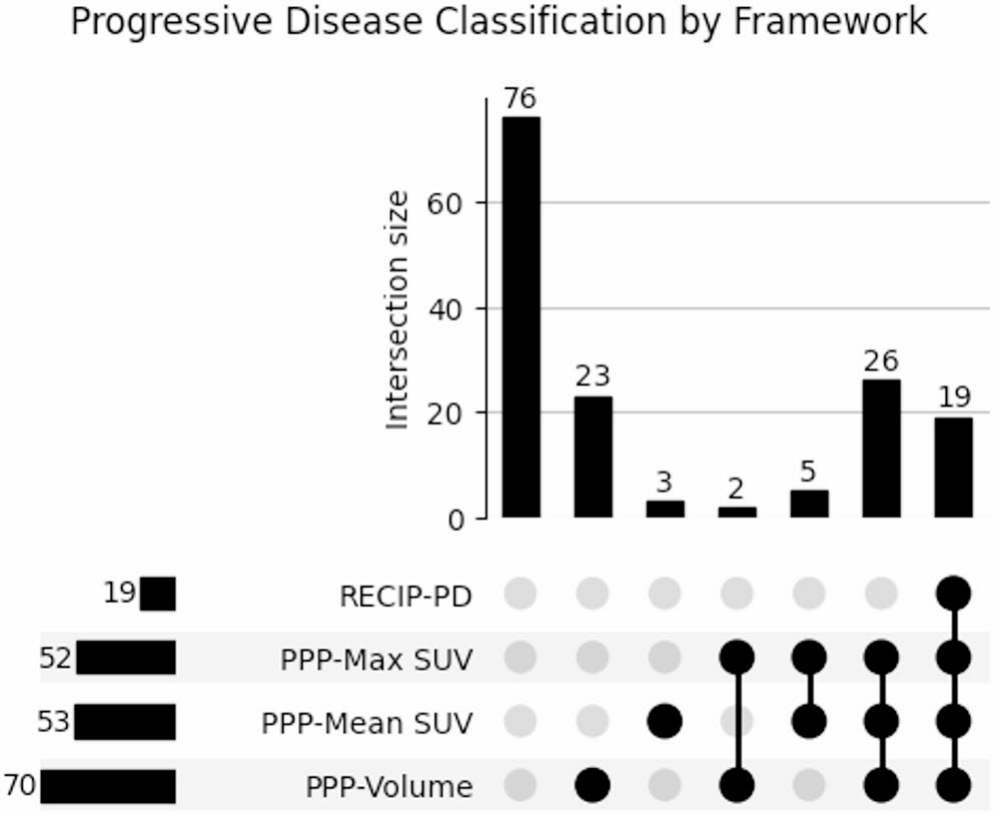
UpSet plot of progressive disease classifications by each framework. Vertical bars show the number of patients meeting each unique combination of criteria; horizontal bars show total progressive disease counts per framework. PPP, PSMA PET Progression; SUV, standardised uptake value; RECIP-PD, Response Evaluation Criteria in PSMA PET/CT–Progressive Disease

Using baseline and follow-up scans, all 154 patients were classified as having progressive disease (PD) or non-PD under the four progression frameworks: PPP-Volume, PPP-Mean SUV, PPP-Max SUV, and RECIP-PD, with classifications represented in Fig. 3. Additionally, patients were categorised into the four RECIP 1.0-defined categories represented in Table 3.

**Table 3:** Number of patients categorised under each RECIP 1.0 category

RECIP Category	Count (*n* = 154)
RECIP-CR	23 (14.9%)
RECIP-PR	43 (27.9%)
RECIP-SD	69 (44.8%)
RECIP-PD	19 (12.3%)

### Overall survival and PSA progression-free survival stratified by RECIP status

**Fig. 4:**
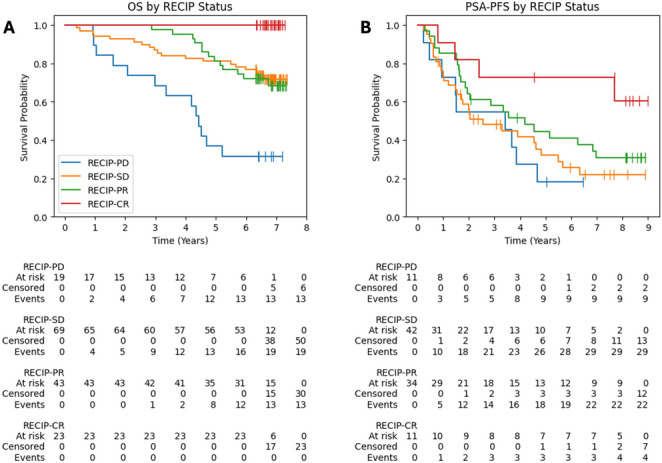
Kaplan-Meier curves for (**A**) overall survival and (**B**) PSA progression-free survival based on RECIP status. Patients “at risk” refers to individuals who were still being followed at each time point and had not yet experienced the event of interest or been censored. OS, overall survival; PSA-PFS, prostate-specific antigen progression-free survival; RECIP 1.0, Response Evaluation Criteria in PSMA PET/CT; RECIP-PD, RECIP progressive disease; RECIP-SD, RECIP stable disease; RECIP-PR, RECIP partial response; RECIP-CR, RECIP complete response

Figure 4 presents Kaplan-Meier analyses of OS and PSA-PFS stratified by RECIP 1.0 classification. Patients classified as RECIP-PD had the poorest OS, with a median of 53.2 months, whereas the median OS was not reached in the non-PD groups. There was no statistically significant difference in survival between the RECIP-SD and RECIP-PR groups (log-rank *p* = 0.87).

RECIP categories did not clearly stratify patients by PSA progression. Median time to progression was shortest in the RECIP-SD group (30.7 months), followed by RECIP-PD (41.1 months), RECIP-PR (50.2 months), and RECIP-CR (not reached). Statistically significant differences in survival distributions were observed between RECIP-PD and RECIP-CR (log-rank *p* = 0.02) and between RECIP-SD and RECIP-CR (log-rank *p* = 0.02) only.

### Overall survival and PSA progression-free survival stratified by PPP, RECIP-PD and PROMISE nomograms

**Fig. 5:**
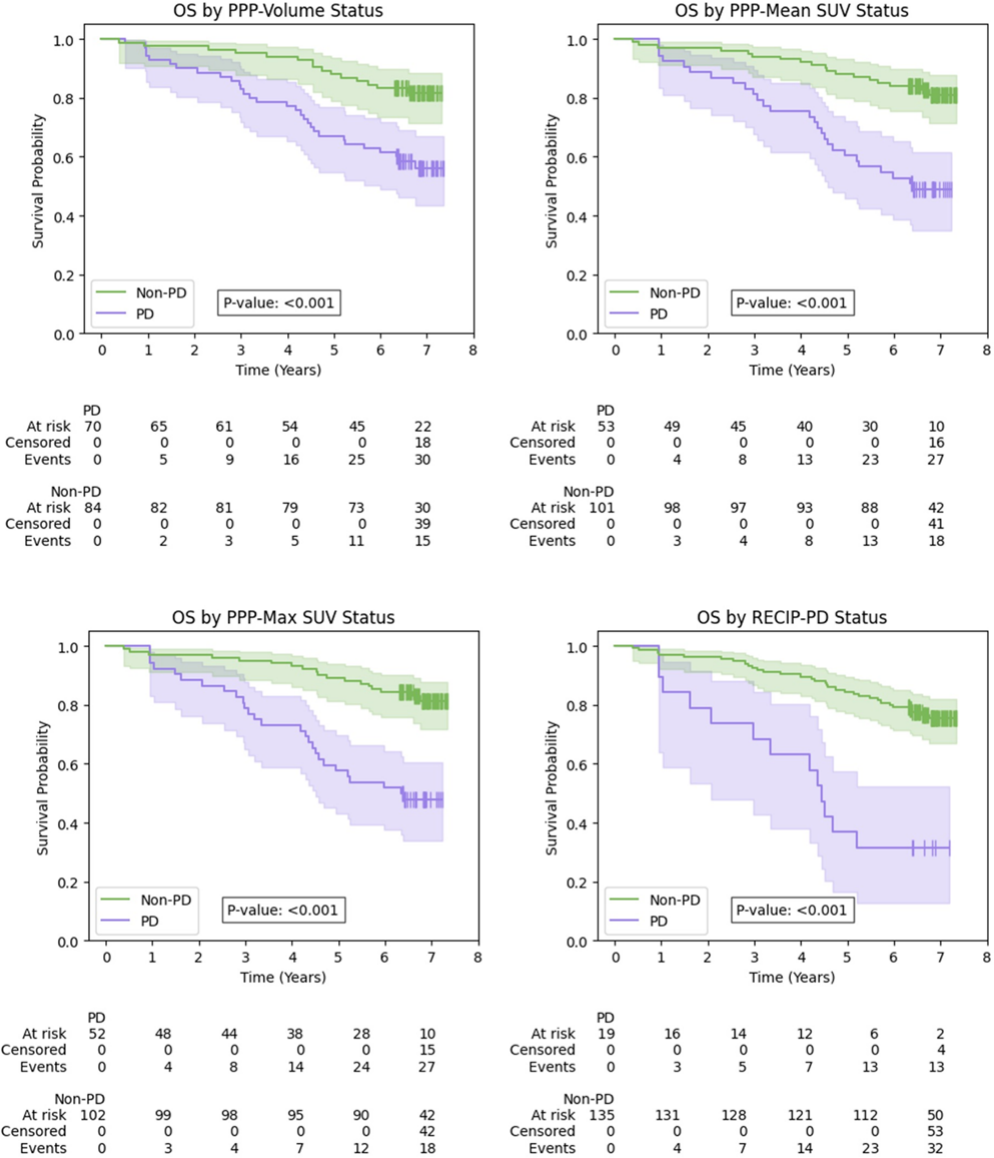
Kaplan-Meier curves for overall survival by response frameworks. OS, overall survival; PD, progressive disease

**Fig. 6:**
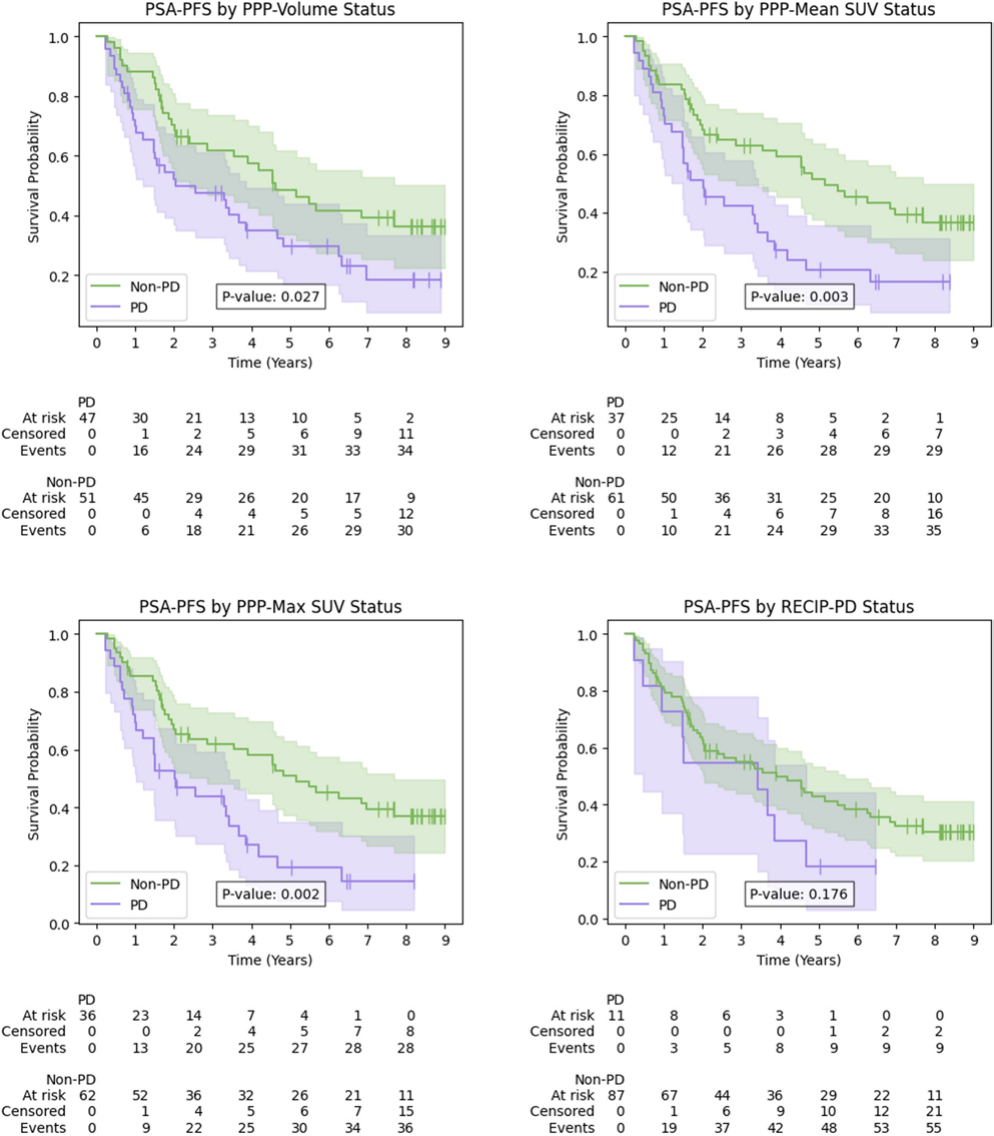
Kaplan-Meier curves for PSA-PFS by response frameworks. PSA-PFS, prostate-specific antigen progression-free survival; PD, progressive disease

A comprehensive summary of results is presented in Table 4, with the corresponding Kaplan-Meier curves for PPP and RECIP in Figs. 5 and 6 respectively. Kaplan-Meier curves for the visual and quantitative PROMISE-based nomograms for OS are found in Supplementary Fig. 1. In the OS analysis, all frameworks stratified patients into PD and non-PD groups (or high- and low-risk groups in the case of the nomograms), with significantly different survival distributions based on log-rank testing. The PD/high-risk groups were consistently associated with increased mortality risk in all frameworks. The RECIP-PD classification identified patients with the most pronounced reduction in survival, with a median OS of 53.2 months, compared to a median OS not reached in the non-PD group (*p* < 0.001).

**Table 4:** Results of Kaplan-Meier and Cox proportional hazards analyses for all criteria in the OS and PSA-PFS endpoints

		Kaplan-Meier analysis			Cox proportional hazards analysis		
		Median time to event (months)					
Endpoint	Progression framework	Non-PD	PD	Log-rank p-value	HR (95% CI)	p-value	C-index
OS (*n* = 154)	PPP-Volume	*	*	< 0.001	2.88 (1.55–5.35)	< 0.001	0.63
	PPP-Mean SUV	*	76.6	< 0.001	3.62 (1.99–6.56)	< 0.001	0.66
	PPP-Max SUV	*	76.0	< 0.001	3.84 (2.11–6.99)	< 0.001	0.67
	RECIP-PD	*	53.2	< 0.001	4.62 (2.41–8.87)	< 0.001	0.61
	Visual PROMISE Nomogram^†^	*	*	< 0.001	2.66 (1.49–4.75)	< 0.001	0.62
	Quantitative PROMISE Nomogram^†^	*	*	< 0.001	3.62 (1.83–7.13)	< 0.001	0.65
PSA-PFS (*n* = 98)	PPP-Volume	55.4	24.4	0.027	1.74 (1.06–2.85)	0.029	0.58
	PPP-Mean SUV	61.7	24.3	0.003	2.09 (1.26–3.45)	0.004	0.59
	PPP-Max SUV	61.7	24.3	0.002	2.19 (1.32–3.64)	0.002	0.59
	RECIP-PD	46.7	41.1	0.176	1.63 (0.80–3.31)	0.179	0.52

†PROMISE-based nomograms stratify patients into high- and low-risk on a single scan; these groups are displayed under the Non-PD/PD columns for ease of comparison only. *Median event time not reached. PD, progressive disease; HR, hazard ratio; CI, confidence interval; OS, overall survival; PSA-PFS, prostate-specific antigen progression-free survival

For the PSA-PFS endpoint, the PPP frameworks were prognostic, with PD patients showing shorter times to progression and higher risk of PSA progression. RECIP-PD did not demonstrate a significant association with PSA-PFS.

### Overall survival and PSA progression-free survival stratified by PPP and RECIP-PD across treatment groups

Analyses were performed for three main treatment groups: ADT only, radiotherapy only, and combined ADT and radiotherapy. Other treatments or combinations were received by six or fewer patients, limiting statistical power for comparison. An UpSet plot of treatment combinations is shown in Supplementary Figs. 2 and 3. Table 5 summarises the analyses, and all Kaplan-Meier curves are shown in Supplementary Figs. 4–9.

**Table 5:** Results of Kaplan-Meier and Cox proportional hazards analyses for all criteria in the overall survival and PSA progression-free survival analyses, stratified by treatment type

ADT patients		Kaplan-Meier analysis			Cox proportional hazards analysis		
		Median time to event (months)					
Endpoint	Progression framework	Non-PD	PD	Log-rank p-value	HR (95% CI)	p-value	C-index
OS (*n* = 65)	PPP-Volume	*	76.6	0.014	2.85 (1.19–6.78)	0.018	0.63
	PPP-Mean SUV	*	62.4	0.002	3.44 (1.53–7.73)	0.003	0.66
	PPP-Max SUV	*	59.3	< 0.001	3.64 (1.62–8.19)	0.002	0.68
	RECIP-PD	*	50.2	< 0.001	5.13 (2.17–12.13)	< 0.001	0.63
PSA-PFS (*n* = 46)	PPP-Volume	23.4	21.2	0.076	1.86 (0.93–3.73)	0.081	0.56
	PPP-Mean SUV	22.3	30.7	0.336	1.41 (0.70–2.86)	0.335	0.50
	PPP-Max SUV	22.3	30.7	0.273	1.48 (0.73–3.00)	0.273	0.52
	RECIP-PD	22.3	44.1	0.979	1.02 (0.39–2.65)	0.975	0.49
Radiotherapy patients							
OS (*n* = 36)	PPP-Volume	*	*	0.061	4.42 (0.81–24.18)	0.087	0.68
	PPP-Mean SUV	*	*	0.003	8.51 (1.55–46.78)	0.014	0.74
	PPP-Max SUV	*	71.8	0.001	10.54 (1.91–58.10)	0.007	0.76
	RECIP-PD	*	54.1	< 0.001	NA^†^	NA^†^	NA^†^
PSA-PFS (*n* = 18)	PPP-Volume	*	56.1	0.165	2.68 (0.63–11.37)	0.182	0.62
	PPP-Mean SUV	82.1	56.1	0.214	2.26 (0.60–8.48)	0.226	0.64
	PPP-Max SUV	82.1	56.1	0.204	2.49 (0.58–10.61)	0.219	0.61
	RECIP-PD	82.1	56.1	0.428	1.90 (0.38–9.46)	0.435	0.55
ADT + radiotherapy patients							
OS (*n* = 34)	PPP-Volume	*	*	0.020	5.45 (1.10–27.21)	0.038	0.71
	PPP-Mean SUV	*	*	0.106	3.08 (0.73–12.91)	0.124	0.64
	PPP-Max SUV	*	*	0.106	3.08 (0.73–12.91)	0.124	0.64
	RECIP-PD	*	40.1	0.127	3.26 (0.65–16.28)	0.149	0.59
PSA-PFS (*n* = 25)	PPP-Volume	55.4	17.8	0.097	2.32 (0.83–6.47)	0.107	0.64
	PPP-Mean SUV	*	14.5	0.001	5.07 (1.75–14.70)	0.003	0.71
	PPP-Max SUV	*	14.5	0.001	5.07 (1.75–14.70)	0.003	0.71
	RECIP-PD	55.4	5.5	0.028	5.06 (1.01–25.34)	0.049	0.56

*Median event time not reached. †Hazard ratio and corresponding p-value and C-index could not be estimated due to quasi-complete separation, where events were heavily concentrated in one group, preventing model convergence. OS, overall survival; PSA-PFS, PSA progression-free survival; PD, progressive disease; HR, hazard ratio; CI, confidence interval; ADT, androgen deprivation therapy

Among ADT-only patients, RECIP-PD was associated with the shortest median survival time (50.2 months) and the highest risk of death (HR: 5.13; 95% CI: 2.17–12.13; *p* < 0.001). In the radiotherapy-only group, RECIP-PD also showed the shortest median survival (54.1 months); however, the Cox model did not converge, likely due to quasi-complete separation where events were heavily concentrated in one group, rendering the risk estimate unquantifiable. In contrast, PPP-Max SUV demonstrated strong prognostic value in radiotherapy patients, with the highest observed risk of death (HR: 10.54; 95% CI: 1.91–58.10; *p* = 0.007) and strongest concordance (C-index: 0.76). For PSA-PFS, no significant differences were observed between PD and non-PD groups in either treatment setting.

Among patients receiving combined ADT and radiotherapy, only PPP-Volume identified a PD group with significantly poorer OS. For PSA-PFS, RECIP-PD showed the shortest time to progression (5.5 months) but had the lowest concordance (C-index: 0.56). In contrast, PPP-Mean SUV and PPP-Max SUV demonstrated stronger prognostic value, each identifying PD groups with a median progression time of 14.5 months, a higher risk of progression (HR: 5.07; 95% CI: 1.75–14.70; *p* = 0.003) and a concordance index of 0.71.

## Discussion

This study evaluated four PSMA PET–based progression frameworks in patients with BCR PCa: PPP-Volume, PPP-Mean SUV, PPP-Max SUV, and RECIP 1.0. Using baseline and follow-up [^68^Ga]Ga-PSMA-11 PET/CT scans, we assessed their predictive value for OS and PSA-PFS endpoints. In this early-stage cohort, PD as defined by all frameworks was associated with poorer survival and higher risk of death. RECIP-PD identified the highest risk (HR: 4.62; 95% CI: 2.41–8.87; *p* < 0.001) and the shortest median OS (53.2 months vs. not reached). All RECIP-PD cases were also PD under PPP criteria, suggesting RECIP is a more selective framework. PROMISE-based nomograms were also effective in stratifying patients into high- and low-risk groups using a single scan. For PSA-PFS, RECIP-PD showed no significant association with progression risk and showed poor discrimination between PD and non-PD groups. These findings indicate that different progression frameworks may be optimal depending on the clinical context.

RECIP 1.0 was introduced by Gafita et al. in 2022 using a cohort of 124 metastatic castration resistant prostate cancer (mCRPC) patients treated with ^177^Lu-PSMA therapy [9]. Importantly, RECIP is intrinsically tied to PSMA PET imaging, and its use in PSMA-targeted treatment settings may inherently favour its prognostic performance. Several studies in similar cohorts have supported its prognostic value [13, 16, 27], including a follow-up study by Gafita et al. reporting stronger risk stratification with RECIP-PD (HR = 4.33) than PPP (HR = 2.72) [12]. However, findings have not been uniform—Sheikh et al. found no OS difference between RECIP-PD and non-PD patients [28]. Before RECIP’s introduction, Michalski et al. had already shown the prognostic utility of PPP in a similar treatment setting [10].

While RECIP-PD appears promising in mCRPC patients receiving ^177^Lu-PSMA therapy, more generalisable insights may come from studies involving non-PSMA-targeted treatments. In a 2023 study of mCRPC patients treated with androgen receptor pathway inhibitors (ARPIs), Shagera et al. found RECIP-PD to be significantly associated with OS (HR = 5.6; 95% CI: 1.69–18.26; *p* = 0.005) [14]. In a cohort receiving radium-223, both RECIP and PPP were prognostic, with similar HRs (2.9 vs. 2.8) and C-indices (0.66 vs. 0.63) [17]. PPP also showed prognostic value in studies by Lunger et al. (chemotherapy-treated metastatic hormone sensitive PCa and mCRPC patients) [11] and Küper et al. (mixed therapies), where RECIP was not prognostic [29]. A systematic review by Mourato et al. (*n* = 516) found RECIP to be prognostic overall, with a subgroup analysis (*n* = 224) suggesting a non-significant trend favouring RECIP over PPP [30]. Kendrick et al. also demonstrated the prognostic value of RECIP in a BCR PCa cohort drawn from the same trial population used in this study [31].

Our findings support RECIP-PD as the strongest predictor of OS, identifying the highest risk of death (HR: 4.62; 95% CI: 2.41–8.87; *p* < 0.001) and shortest median survival. PPP-based criteria were also prognostic, with all frameworks showing similar concordance ranging from 0.61 to 0.67.

The PROMISE-based nomograms offer a practical single-scan alternative for risk stratification. In our BCR cohort, both the visual and quantitative models effectively separated high- and low-risk groups, with the visual nomogram yielding a HR of 2.66 (95% CI: 1.49–4.75; *p* < 0.001) and a C-index of 0.62, and the quantitative nomogram yielding a HR of 3.62 (95% CI: 1.83–7.13; *p* < 0.001) and a C-index of 0.65. These findings are encouraging given that only a single scan is required. This point-in-time approach may be particularly valuable in settings where follow-up imaging is limited—such as in the absence of public funding for serial PET scans or where out-of-pocket costs restrict access. Unlike RECIP and PPP, which assess treatment response using baseline and follow-up scans, PROMISE provides point-in-time risk stratification based on a single routine scan. As such, comparisons between these approaches should be interpreted with their differing purposes in mind.

Fewer studies have analysed the relationship between progression frameworks and PSA-PFS, although nearly half of PCa trials use it as a surrogate endpoint for OS [32]. However, large-scale evidence has shown that PSA-PFS is not a valid substitute [33]. Nonetheless, it remains a clinically important endpoint for evaluating short-term treatment effects. In our study, PPP frameworks were prognostic for PSA-PFS, whereas RECIP-PD was not. The survival distribution for RECIP-PD also did not differ significantly from RECIP-PR or RECIP-SD. This contrasts with findings by Gafita et al., who observed significantly shorter PSA-PFS in RECIP-PD patients within an advanced disease cohort [15]. The discrepancy may reflect differences in disease stage and progression definitions—PPP identifies progression per lesion, while RECIP captures overall tumour burden and the appearance of new lesions. PSA-PFS, occurring on a shorter timescale, may be more sensitive to subtle or diffuse disease changes missed by RECIP. These findings suggest PPP may be better suited to PSA-PFS prognostication, particularly in early-stage disease or non-PSMA-based treatment settings.

In the ADT-only subgroup, RECIP-PD identified the shortest median OS and highest HR among all frameworks. This supports the hypothesis that RECIP is better suited to systemic therapies, which target disease diffusely. By defining progression based on new lesions or increased overall tumour burden, RECIP offers a whole-body measure aligned with ADT’s mechanism of action. In contrast, PPP frameworks assess lesion-level changes, which may not fully reflect clinically meaningful progression in this context. These findings suggest RECIP provides more relevant prognostic information for patients receiving ADT. None of the frameworks were prognostic for PSA-PFS in this subgroup.

In the radiotherapy-treated subgroup, RECIP-PD again showed the shortest median OS, but the Cox model did not converge—likely due to few events and quasi-complete separation. In contrast, PPP-Max SUV was strongly prognostic, with the highest HR (10.54; 95% CI: 1.91–58.10; *p* = 0.007) and best discriminative performance (C-index: 0.76). As radiotherapy is a focal treatment, lesion-level measures like PPP-Max SUV may better capture local responses or recurrence. This metric focuses on the most PSMA-intense regions within lesions, making it potentially more sensitive to radiotherapy effects than RECIP, which tracks global disease progression. None of the frameworks were prognostic for PSA-PFS in this subgroup.

Among patients treated with both ADT and radiotherapy, only PPP-Volume was prognostic for OS, with a relatively high concordance index of 0.71. This may reflect its sensitivity to gross volumetric changes across both local and systemic disease sites. In this combined setting, PPP-Volume may better capture the complementary effects of systemic and focal therapies than frameworks relying solely on uptake thresholds. RECIP, which requires both new lesions and a ≥ 20% volume increase, may be too conservative—missing progression in patients with rising tumour burden but no new lesions. SUV-based PPP criteria may also be affected by treatment-induced suppression of PSMA uptake, potentially masking active disease [34].

For PSA-PFS in this subgroup, RECIP-PD showed the shortest time to progression but weak discriminative ability (C-index: 0.56). PPP-Mean SUV and PPP-Max SUV had stronger performance, with higher HRs (5.07; 95% CI: 1.75–14.70; *p* = 0.003) and concordance (0.71). This suggests SUV-based PPP criteria may better detect early changes in PSMA uptake associated with PSA progression. PPP-Volume was not prognostic, likely because volumetric changes lag behind biological activity, while SUV metrics are more responsive to early shifts in tumour behaviour.

While this study provides valuable insights into the prognostic utility of RECIP 1.0, PPP and PROMISE-based nomograms in early-stage PCa, it has several limitations. The retrospective design introduces potential bias, particularly in patient selection and treatment heterogeneity, as patients received different initial therapies and management strategies. The timing between baseline and follow-up PSMA PET/CT scans also varied considerably (range: 3.2–8.8 months) introducing temporal variability. The small sample sizes in treatment-specific subgroups reduced statistical power for stratified analyses. Disease progression was also assessed without biopsy confirmation, relying solely on imaging and PSA, which may not fully capture biological activity. Additionally, the effect of recent treatment on PSMA uptake is not fully understood. Emerging evidence suggests that different therapies, and the timing of imaging relative to those therapies, can alter PSMA expression in ways that do not always align with PSA levels or other biomarkers [34–36]. Future prospective studies could address these limitations by standardising imaging timepoints and stratifying patients by initial definitive treatment prior to analysis.

Despite these limitations, our findings have important clinical implications. RECIP-PD consistently demonstrated the strongest prognostic value for OS, particularly among patients receiving systemic therapies such as ADT, where its whole-body approach may better reflect diffuse disease progression. Its higher specificity may also help avoid overtreatment by more reliably identifying those who require early intervention. In contrast, PPP-based frameworks, which assess lesion-level changes, showed greater utility for short-term endpoints such as PSA progression-free survival, and may be more suitable for monitoring response in patients receiving localised treatments like radiotherapy. These results support a more personalised approach to progression assessment, aligning the choice of criteria with treatment context and the specific clinical endpoint.

## Conclusion

This study assessed PSMA PET–based progression criteria in BCR PCa, comparing three PPP definitions to RECIP 1.0. RECIP-PD was most strongly associated with poorer outcomes in the OS endpoint, while PPP-based criteria demonstrated better prognostic performance for PSA-PFS. These findings suggest that RECIP is better suited to long-term risk stratification, whereas PPP frameworks may offer greater utility for monitoring short-term biochemical progression. Further prospective studies are warranted to validate these findings and refine the clinical application of RECIP 1.0 and PPP in BCR management.

## Supplementary Information

Below is the link to the electronic supplementary material.

Supplementary Material 1 (DOCX 3.61 MB)

## Data Availability

The datasets analysed during the current study are not publicly available due to patient privacy restrictions.

## References

[1] Bray F, et al. Global cancer statistics 2022: GLOBOCAN estimates of incidence and mortality worldwide for 36 cancers in 185 countries. CA Cancer J Clin. 2024;74(3):229–63. 38572751 10.3322/caac.21834

[2] Simon NI, et al. Best approaches and updates for prostate cancer biochemical recurrence. Am Soc Clin Oncol Educational Book. 2022;42:352–9. 10.1200/EDBK_351033 PMC9844546 35503984

[3] Tourinho-Barbosa R, et al. Biochemical recurrence after radical prostatectomy: what does it mean? Int Braz J Urol. 2018;44:14–21. 29039897 10.1590/S1677-5538.IBJU.2016.0656 PMC5815528

[4] Scher HI, et al. Trial design and objectives for castration-resistant prostate cancer: updated recommendations from the prostate cancer clinical trials working group 3. J Clin Oncol. 2016;34(12):1402–18. 26903579 10.1200/JCO.2015.64.2702 PMC4872347

[5] Collette L, Burzykowski T, Schröder FH. Prostate-specific antigen (PSA) alone is not an appropriate surrogate marker of long-term therapeutic benefit in prostate cancer trials. Eur J Cancer. 2006;42(10):1344–50. 16730974 10.1016/j.ejca.2006.02.011

[6] Afshar-Oromieh A, et al. Diagnostic performance of 68 Ga-PSMA-11 (HBED-CC) PET/CT in patients with recurrent prostate cancer: evaluation in 1007 patients. Eur J Nucl Med Mol Imaging. 2017;44:1258–68. 28497198 10.1007/s00259-017-3711-7 PMC5486817

[7] Lenzo NP, Meyrick D, Turner JH. Review of gallium-68 PSMA PET/CT imaging in the management of prostate cancer. Diagnostics. 2018;8(1):16. 29439481 10.3390/diagnostics8010016 PMC5871999

[8] Fanti S, Hadaschik B, Herrmann K. *Proposal for systemic-therapy response-assessment criteria at the time of PSMA PET/CT imaging: the PSMA PET progression criteria*. 2020, Soc Nuclear Med. pp. 678–682. 10.2967/jnumed.119.233817 PMC7198387 31806774

[9] Gafita A, et al. Novel framework for treatment response evaluation using PSMA PET/CT in patients with metastatic castration-resistant prostate cancer (RECIP 1.0): an international multicenter study. J Nucl Med. 2022;63(11):1651–8. 35422442 10.2967/jnumed.121.263072 PMC9635677

[10] Michalski K, et al. Assessing response to 177Lu-PSMA radioligand therapy using modified PSMA PET progression criteria. J Nucl Med. 2021;62(12):1741–6. 33789932 10.2967/jnumed.120.260836 PMC8612188

[11] Lunger L, et al. Prognostic role of 68Ga-PSMA11 PET–based response in patients with prostate cancer undergoing taxane-based chemotherapy. J Nucl Med. 2023;64(6):896–901. 36581373 10.2967/jnumed.122.264962

[12] Gafita A, et al. Measuring response in metastatic castration-resistant prostate cancer using PSMA PET/CT: comparison of RECIST 1.1, aPCWG3, aPERCIST, PPP, and RECIP 1.0 criteria. Eur J Nucl Med Mol Imaging. 2022;49(12):4271–81. 35767071 10.1007/s00259-022-05882-x

[13] Kind F, et al. Prognostic value of tumor volume assessment on PSMA PET after ^177^Lu-PSMA radioligand therapy evaluated by PSMA PET/CT consensus statement and RECIP 1.0. J Nucl Med. 2023;64(4):605–10. 36302658 10.2967/jnumed.122.264489

[14] Shagera QA, et al. PSMA PET/CT for response assessment and overall survival prediction in patients with metastatic castration-resistant prostate cancer treated with androgen receptor pathway inhibitors. J Nucl Med. 2023;64(12):1869–75. 37770114 10.2967/jnumed.123.265874

[15] Gafita A, et al. RECIP 1.0 predicts progression-free survival after [177Lu] Lu-PSMA radiopharmaceutical therapy in patients with metastatic castration-resistant prostate cancer. J Nucl Med. 2024;65(6):917–22. 38637143 10.2967/jnumed.123.267234

[16] Murthy V, et al. Prognostic value of end-of-treatment PSMA PET/CT in patients treated with ^177^Lu-PSMA radioligand therapy: a retrospective, single-center analysis. J Nucl Med. 2023;64(11):1737–43. 37678927 10.2967/jnumed.122.265155

[17] Shagera QA, et al. Evaluating response to radium-223 using ^68^Ga-PSMA PET/CT imaging in patients with metastatic castration-resistant prostate cancer. Ann Nucl Med. 2025;39(2):208–16. 39368051 10.1007/s12149-024-01990-w

[18] Karpinski MJ, et al. Combining PSMA-PET and PROMISE to re-define disease stage and risk in patients with prostate cancer: a multicentre retrospective study. Lancet Oncol. 2024;25(9):1188–201. 39089299 10.1016/S1470-2045(24)00326-7

[19] Eiber M, et al. Prostate cancer molecular imaging standardized evaluation (PROMISE): proposed MiTNM classification for the interpretation of PSMA-ligand PET/CT. J Nucl Med. 2018;59(3):469–78. 29123012 10.2967/jnumed.117.198119

[20] McCarthy M, et al. A multicenter prospective clinical trial of 68Gallium PSMA HBED-CC PET-CT restaging in biochemically relapsed prostate carcinoma: oligometastatic rate and distribution compared with standard imaging. Int J Radiat Oncol Biol Phys. 2019;104(4):801–8. 30890448 10.1016/j.ijrobp.2019.03.014

[21] Francis RJ, et al. The australasian radiopharmaceutical trials network: clinical trials, evidence, and opportunity. J Nucl Med. 2021;62(6):755. 33384324 10.2967/jnumed.120.258152 PMC8729868

[22] Ceci F, et al. E-PSMA: the EANM standardized reporting guidelines v1.0 for PSMA-PET. Eur J Nucl Med Mol Imaging. 2021;48:1626–38. 33604691 10.1007/s00259-021-05245-y PMC8113168

[23] Aksu A, Kaya GÇ. *Is SUV* corrected for lean body mass superior to *SUV* of body weight in ^68^Ga-PSMA PET/CT? Mol Imaging Radionucl Ther. 2021;30(3):144. 34658229 10.4274/mirt.galenos.2021.59254 PMC8522520

[24] Gafita A, et al. Evaluation of SUV normalized by lean body mass (SUL) in 68 Ga-PSMA11 PET/CT: a bi-centric analysis. EJNMMI Res. 2019;9:1–6. 31792771 10.1186/s13550-019-0572-z PMC6889088

[25] Zwezerijnen GJ, et al. Reproducibility of [18F] FDG PET/CT liver SUV as reference or normalisation factor. Eur J Nucl Med Mol Imaging. 2023;50(2):486–93. 36166080 10.1007/s00259-022-05977-5 PMC9816285

[26] Davidson-Pilon C. Lifelines: survival analysis in python. J Open Source Softw. 2019;4(40):1317.

[27] Hartrampf PE, et al. Prognostic performance of RECIP 1.0 based on [18F] PSMA-1007 PET in prostate cancer patients treated with [177Lu] Lu-PSMA I&T. J Nucl Med. 2024;65(4):560–5. 38453363 10.2967/jnumed.123.266702

[28] Sheikh GT, et al. RECIP 1.0 + PSA for response assessment in mCRPC patients treated with 225Ac/177Lu PSMA combination therapy. EJNMMI Res. 2025;15(1):19. 40038098 10.1186/s13550-025-01211-z PMC11880445

[29] Küper AT, et al. PSMA-PET follow-up to assess response in patients not receiving PSMA therapy: is there value beyond localization of disease? Theranostics. 2024;14(9):3623. 38948055 10.7150/thno.96738 PMC11209722

[30] Mourato FA, et al. Prognostic value of response evaluation using PSMA PET/CT in patients with metastatic prostate cancer (RECIP 1.0): a systematic review and meta-analysis. Acad Radiol. 2025. 10.1016/j.acra.2024.12.027. 39755494 10.1016/j.acra.2024.12.027

[31] Kendrick J, et al. Prognostic utility of RECIP 1.0 with manual and AI-based segmentations in biochemically recurrent prostate cancer from [68Ga] Ga-PSMA-11 PET images. Eur J Nucl Med Mol Imaging. 2023;50(13):4077–86. 37550494 10.1007/s00259-023-06382-2 PMC10611879

[32] Maeda H, et al. Searching for potential surrogate endpoints of overall survival in clinical trials for patients with prostate cancer. Cancer Rep. 2021;4(3):e1334. 10.1002/cnr2.1334 PMC8222553 33455091

[33] Gharzai LA, et al. Meta-analysis of candidate surrogate end points in advanced prostate cancer. NEJM Evid. 2023;2(4):EVIDoa2200195. 38320030 10.1056/EVIDoa2200195

[34] Hotta M, et al. Kinetics of PSMA PET signal after radiotherapy in prostate cancer lesions: A single-center retrospective study. Radiother Oncol. 2025;207:110869. 40122284 10.1016/j.radonc.2025.110869

[35] Vaz S, et al. Influence of androgen deprivation therapy on PSMA expression and PSMA-ligand PET imaging of prostate cancer patients. Eur J Nucl Med Mol Imaging. 2020;47(1):9–15. 31654093 10.1007/s00259-019-04529-8

[36] Yadav S, et al. Pre-or post-chemotherapy: effect on PSMA uptake. EJNMMI Res. 2025;15(1):36. 40192880 10.1186/s13550-025-01229-3 PMC11977060

