# Supplementary material for: RECIP 1.0 more predictive of overall survival than PSMA PET progression criteria in biochemically recurrent prostate cancer
Source: Eur J Nucl Med Mol Imaging. 2025 Oct 28;53(4):2234–44. doi: 10.1007/s00259-025-07592-6 (PMC12920775; doi:10.1007/s00259-025-07592-6)
Supplement: Supplementary file 1 — Supplementary Material 1 (DOCX 3.61 MB) [file 259_2025_7592_MOESM1_ESM.docx]

RECIP 1.0 More Predictive of Overall Survival Than PSMA PET Progression Criteria in Biochemically Recurrent Prostate Cancer

Kaylee Molin^1,2*^, Jeremy S.L Ong^3^, Steven Van Der Werf^4^, Roslyn J. Francis^5,6,7,8^, Ghulam Mubashar Hassan^1^, Martin A. Ebert^1,2,4^, Jake Kendrick^1,2^

^1^ School of Physics, Mathematics and Computing, University of Western Australia, Crawley, WA, Australia

^2^ Centre for Advanced Technologies in Cancer Research (CATCR), Perth, WA, Australia

^3^ Department of Nuclear Medicine, Fiona Stanley Hospital, Murdoch, WA, Australia

^4^ Department of Radiation Oncology, Sir Charles Gairdner Hospital, Nedlands, WA, Australia

^5^ Department of Nuclear Medicine, Sir Charles Gairdner Hospital, Nedlands, WA, Australia

^6^ Medical School, University of Western Australia, Crawley, WA, Australia

^7^ Department of Nuclear Medicine, Royal Brisbane and Women’s Hospital, Brisbane, QLD, Australia

^8^ Australian Institute for Bioengineering and Nanotechnology, University of Queensland, Brisbane, QLD, Australia

*Corresponding author. E-mail: [kaylee.molin@research.uwa.edu.au](mailto:kaylee.molin@research.uwa.edu.au)

# Supplementary Data


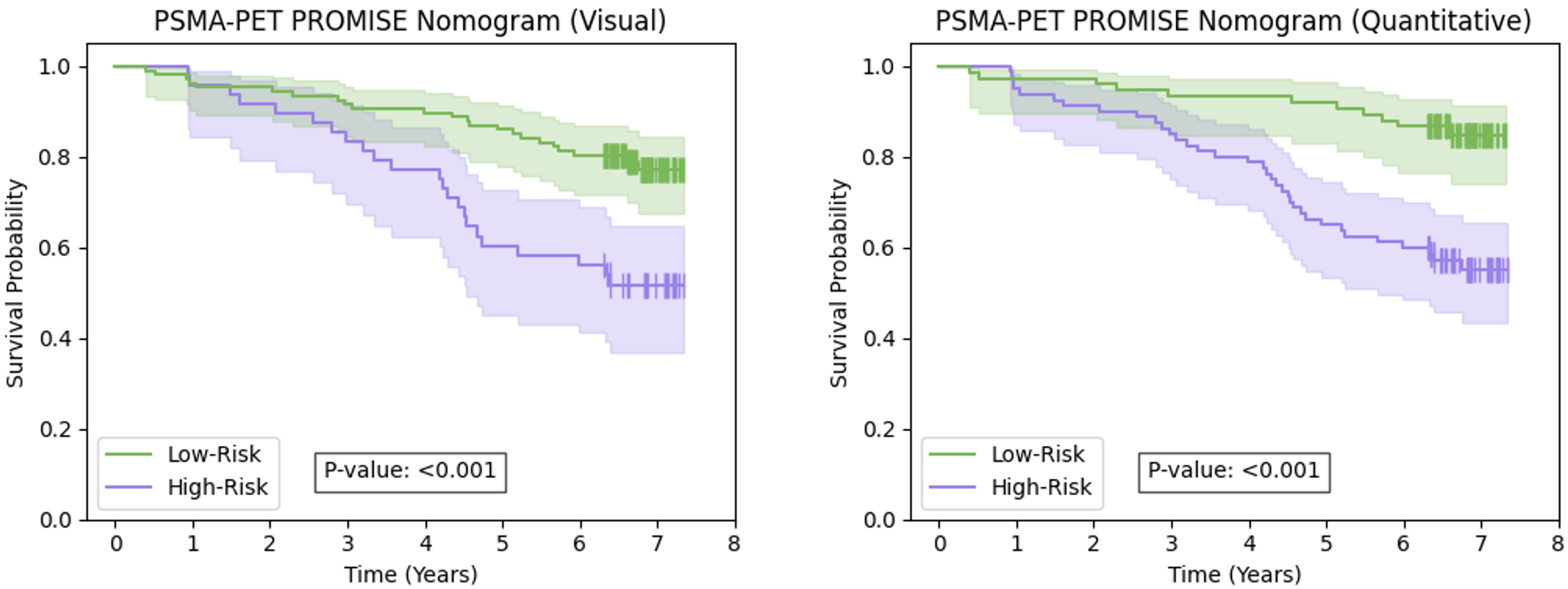


Supplementary Figure 1 Patients stratified into high- and low-risk groups based on the PROMISE-based visual and quantitative nomograms, using the follow-up PSMA PET/CT scan


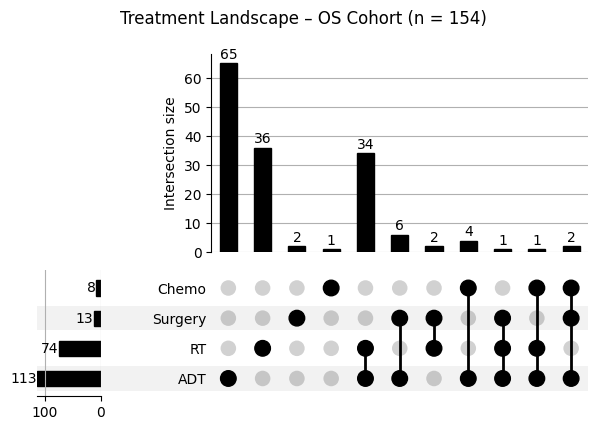


Supplementary Figure 2 UpSet plot of treatment combinations among the 154 patients in the overall survival cohort. RT, radiotherapy; ADT, androgen deprivation therapy


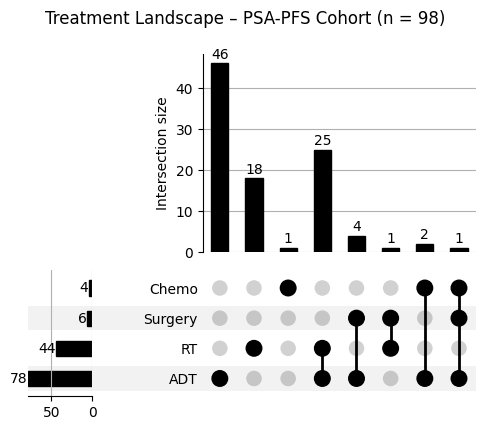


Supplementary Figure 3 UpSet plot of treatment combinations among the 98 patients in the PSA-progression free survival cohort. RT, radiotherapy; ADT, androgen deprivation therapy


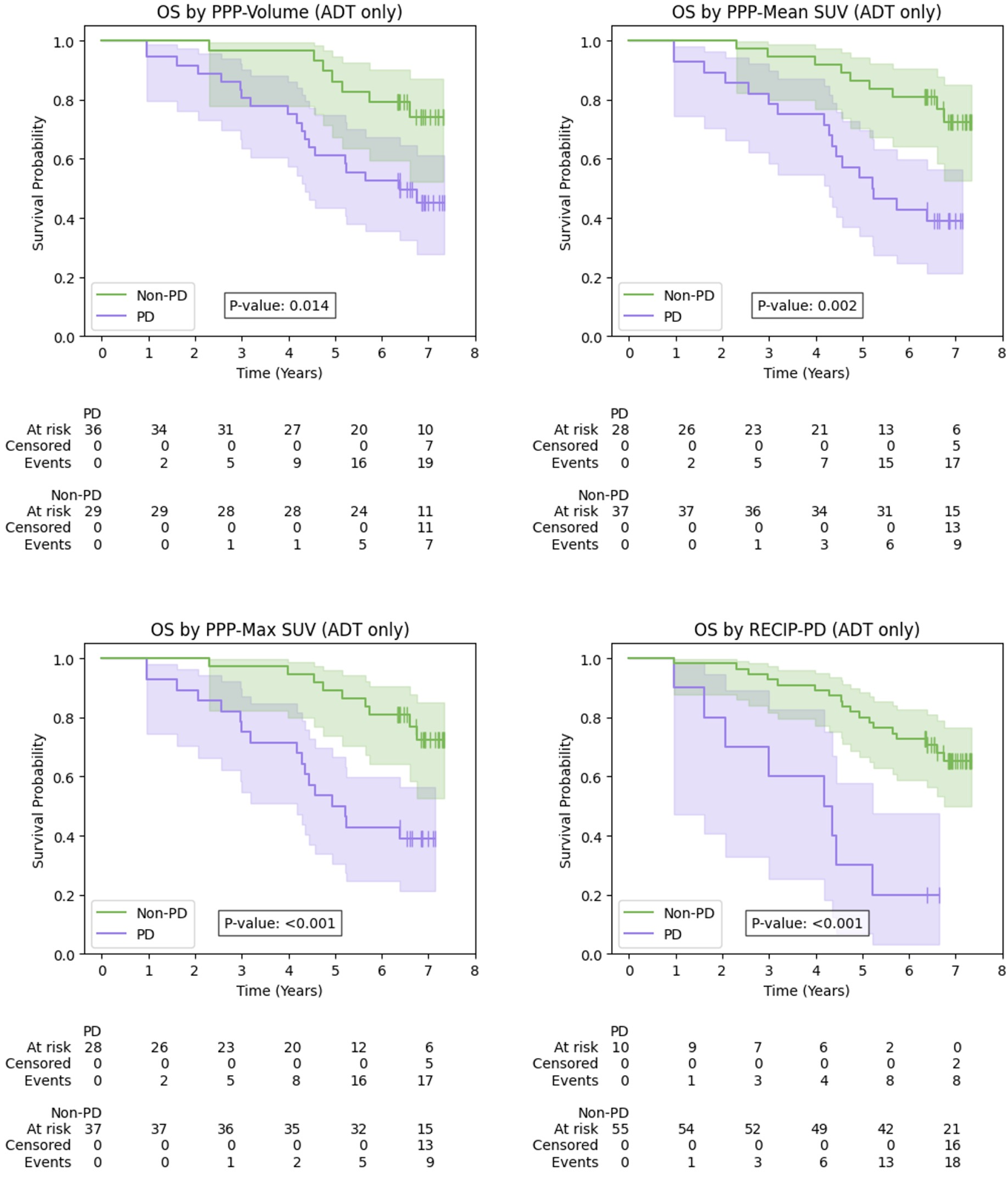


Supplementary Figure 4 Kaplan-Meier curves for overall survival by response frameworks for ADT patients. OS, overall survival; PD, progressive disease; ADT, androgen deprivation therapy


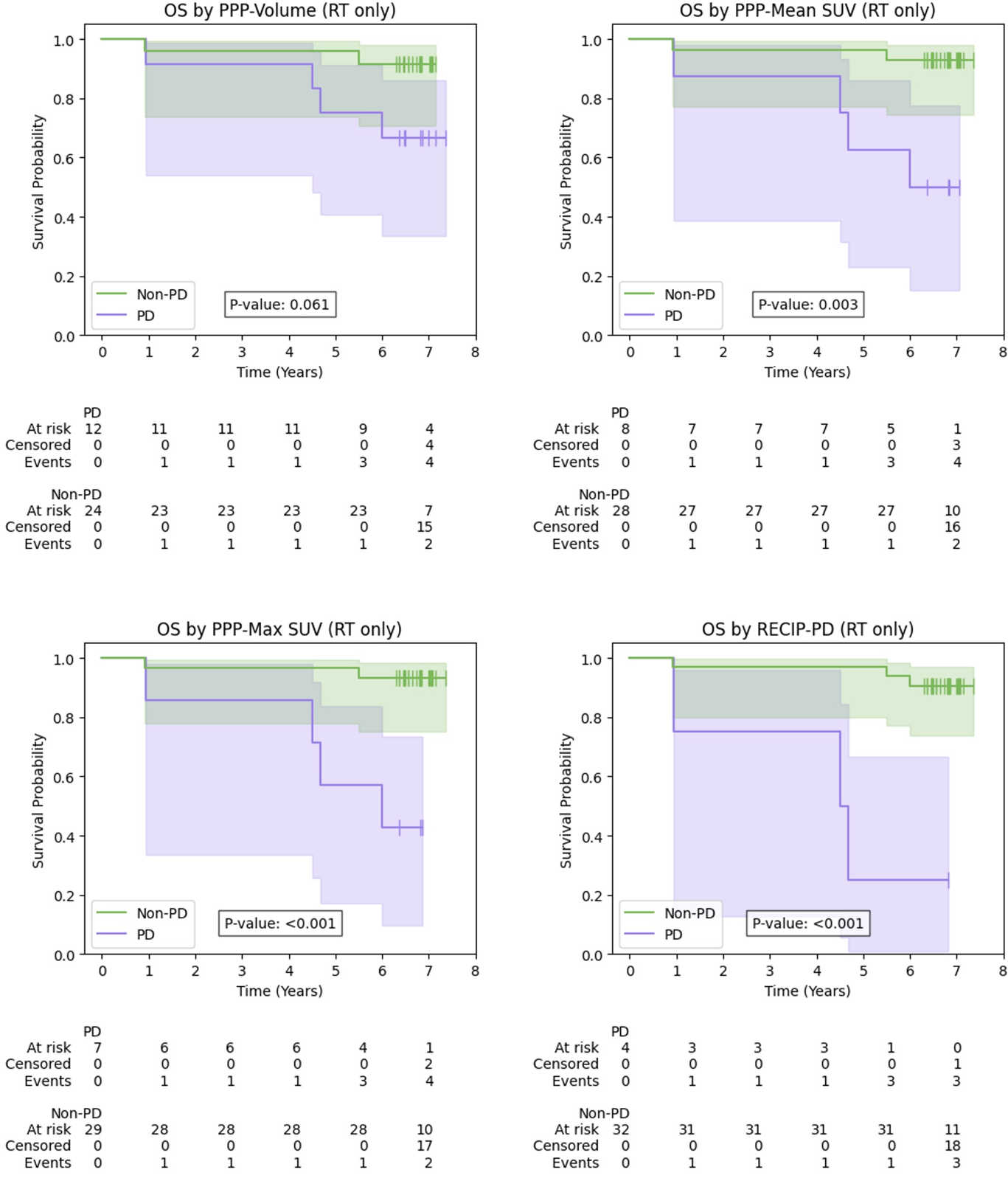


Supplementary Figure 5 Kaplan-Meier curves for overall survival by response frameworks for radiotherapy patients. OS, overall survival; PD, progressive disease; RT, radiotherapy


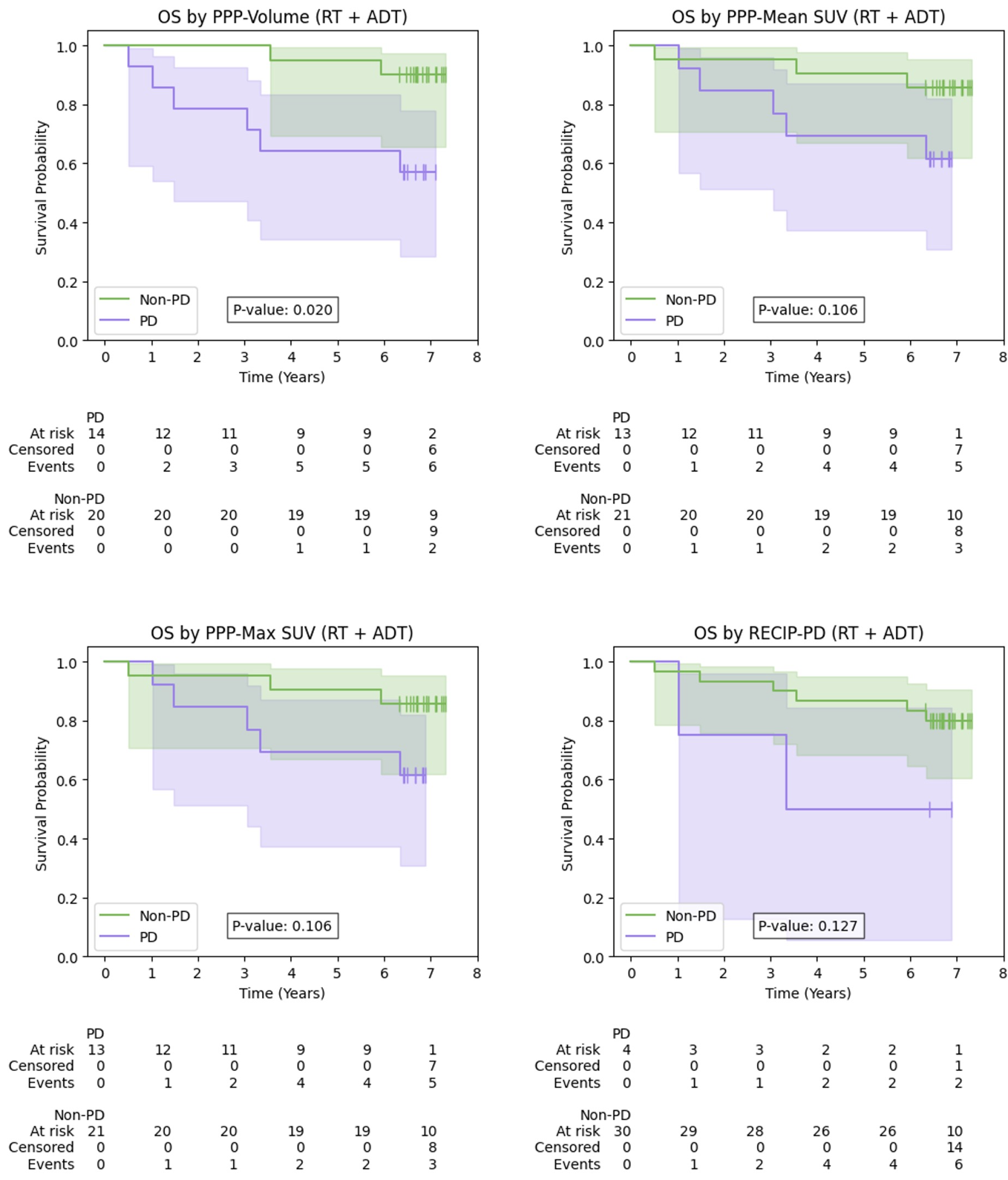


Supplementary Figure 6 Kaplan-Meier curves for overall survival by response frameworks for combination ADT and radiotherapy patients. OS, overall survival; PD, progressive disease; RT, radiotherapy; ADT, androgen deprivation therapy


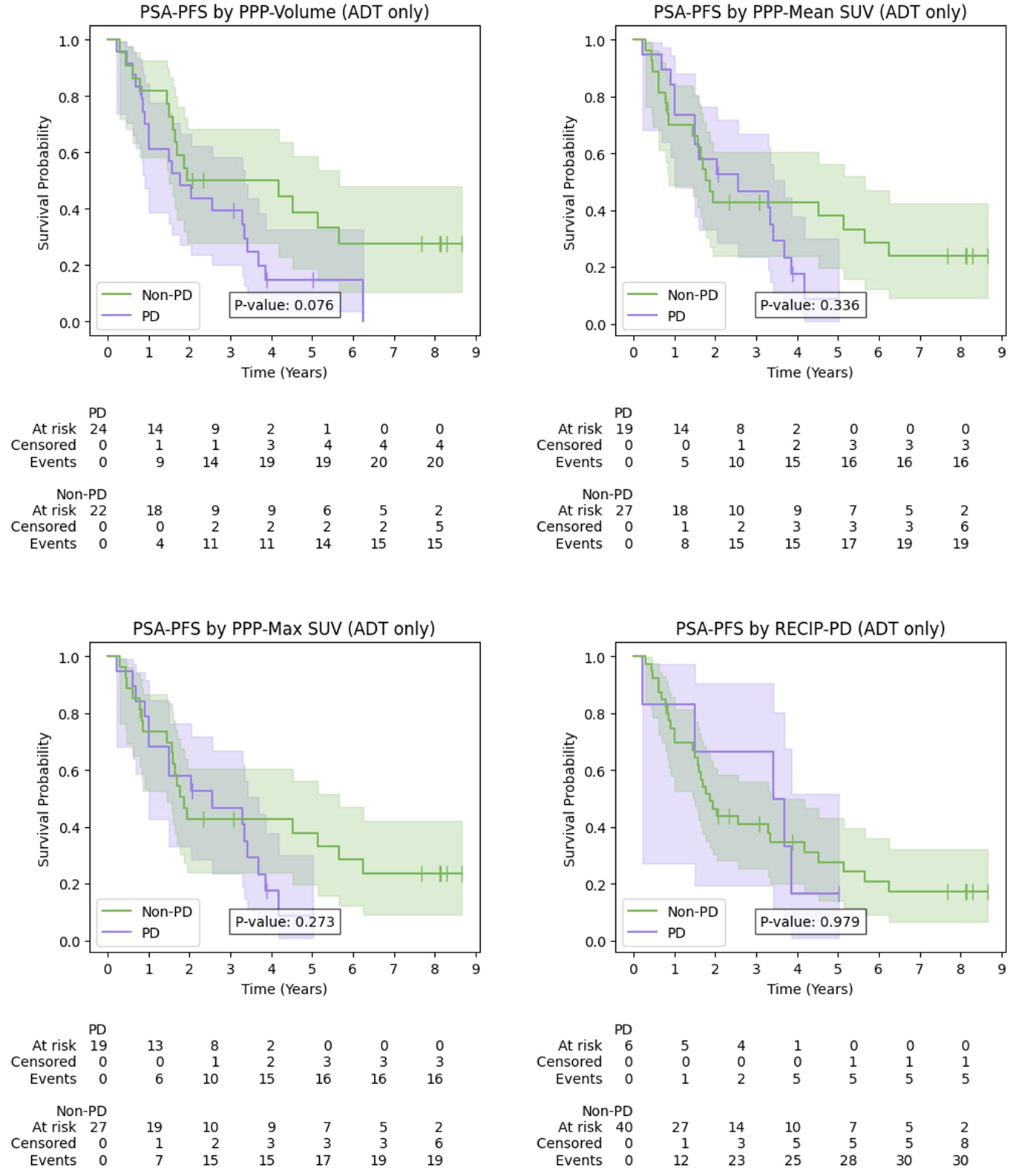


Supplementary Figure 7 Kaplan-Meier curves for PSA-PFS by response frameworks for ADT patients. PSA-PFS, prostate specific antigen progression-free survival; PD, progressive disease; ADT, androgen deprivation therapy


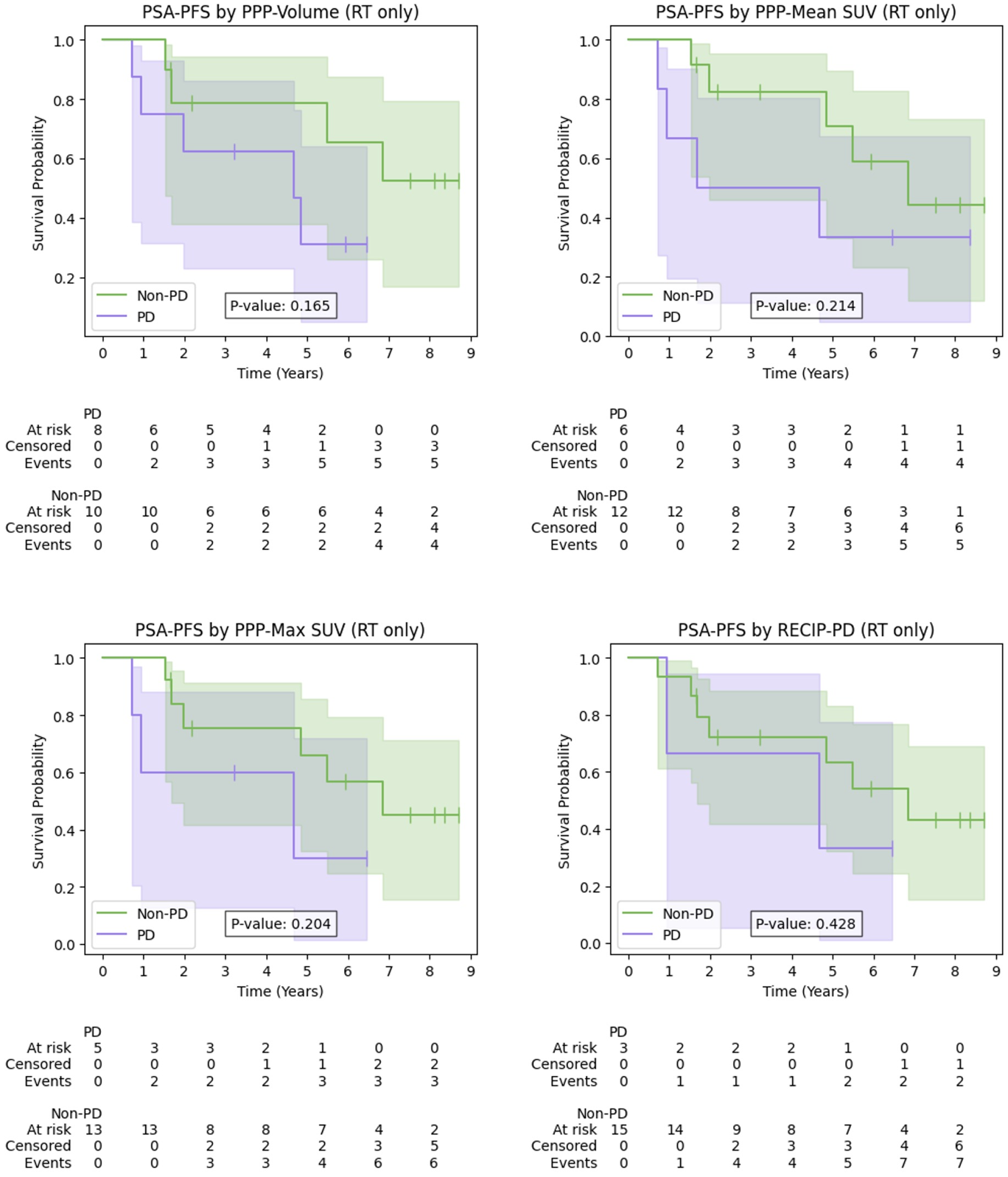


Supplementary Figure 8 Kaplan-Meier curves for PSA-PFS by response frameworks for RT patients. PSA-PFS, prostate specific antigen progression-free survival; PD, progressive disease; RT, radiotherapy


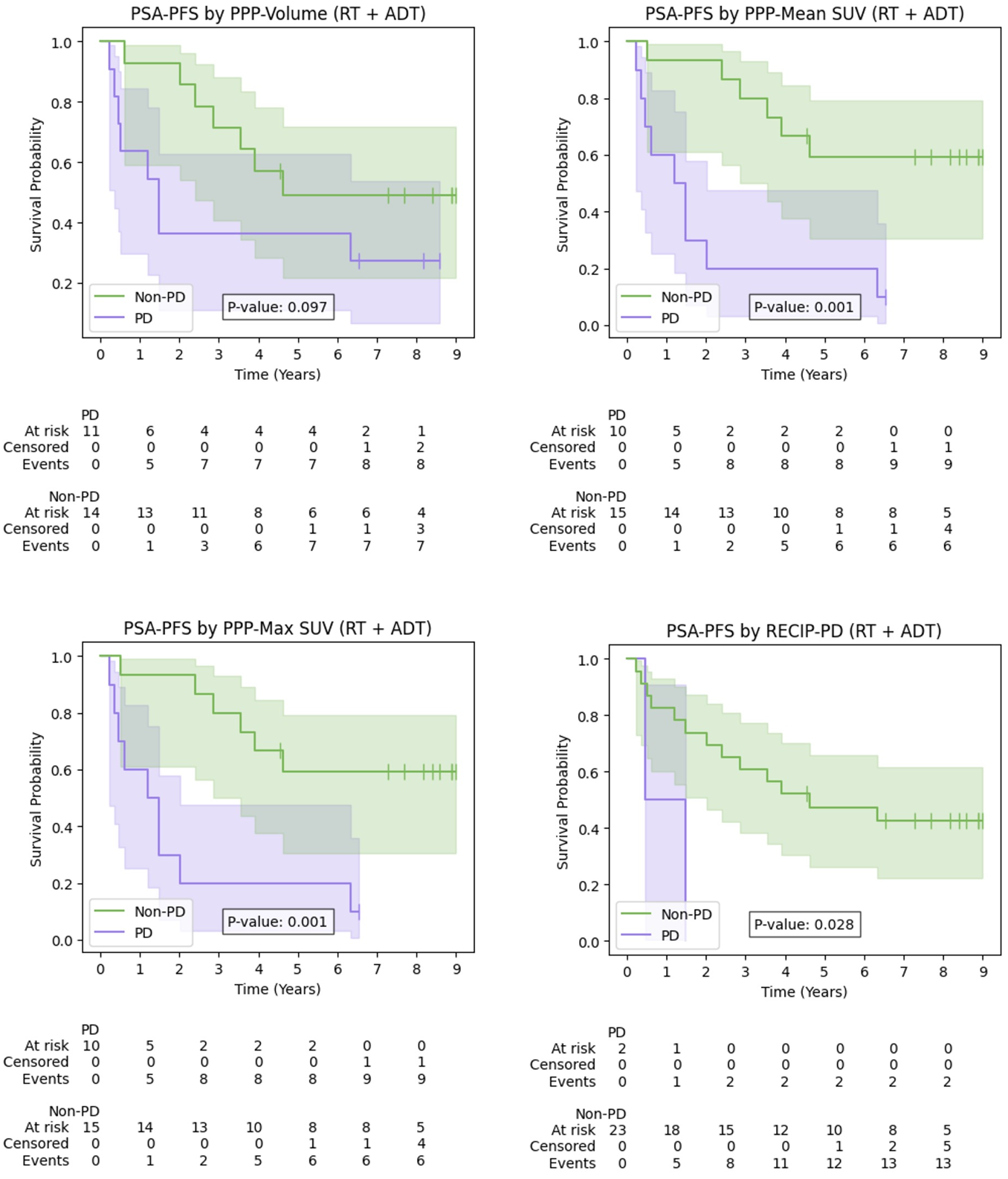


Supplementary Figure 9 Kaplan-Meier curves for PSA-PFS by response frameworks for combination ADT and radiotherapy patients. PSA-PFS, prostate specific antigen progression-free survival; PD, progressive disease; RT, radiotherapy; ADT, androgen deprivation therapy
